# Enhancement of biomass productivity and biochemical composition of alkaliphilic microalgae by mixotrophic cultivation using cheese whey for biofuel production

**DOI:** 10.1007/s11356-024-33877-8

**Published:** 2024-06-17

**Authors:** Ahmed Mohamed Youssef, Mohamed Gomaa, Abdel Kareem S. H. Mohamed, Abdel-Rahim A. El-Shanawany

**Affiliations:** 1 Department of Botany and Microbiology, Faculty of Science, Al-Azhar University, Assiut, 71524 Egypt; 2 Botany & Microbiology Department, Faculty of Science, Assiut University, Assiut, 71516 Egypt

**Keywords:** Alkaliphilic algae, Response surface methodology, Mixotrophic, Soluble carbohydrates, Total protein, Nitrogen deficiency, Biodiesel

## Abstract

The growth of microalgae under alkaline conditions ensures an ample supply of CO_2_ from the atmosphere, with a low risk of crashing due to contamination and predators. The present study investigated the mixotrophic cultivation of two alkaliphilic microalgae (*Tetradesmus obliquus* and *Cyanothece* sp.) using cheese whey as an organic carbon source. The variation in cheese whey concentration (0.5–4.5% (v/v)), culture pH (7–11), and NaNO_3_ concentrations (0–2 gL^−1^) was evaluated using central composite design in response to biomass productivity and the contents of lipids, total proteins, and soluble carbohydrates. Both investigated microalgae effectively utilized cheese whey as an organic carbon source. The optimum conditions for simultaneously maximizing biomass and lipid productivity in *T. obliquus* were 3.5% (v/v) whey, pH 10.0, and 0.5 g L^−1^ NaNO_3_. Under these conditions, the biomass, lipid, soluble carbohydrate, and protein productivities were 48.69, 20.64, 7.02, and 10.97 mg L^−1^ day^−1^, respectively. Meanwhile, *Cyanothece* produced 52.78, 11.42, 4.31, and 7.89 mg L^−1^ day^−1^ of biomass, lipid, carbohydrate, and protein, respectively, at 4.5% (v/v) whey, pH 9.0, and 1.0 g L^−1^ NaNO_3_. The lipids produced under these conditions were rich in saturated fatty acids (FAs) and monounsaturated FAs, with no polyunsaturated FAs in both microalgae. Moreover, several biodiesel characteristics were estimated, and results fell within the ranges specified by international standards. These findings indicate that the mixotrophic cultivation of alkaliphilic microalgae could open new avenues for promoting microalgae productivity through low-cost biofuel production.

**Supplementary Information:**

The online version contains supplementary material available at 10.1007/s11356-024-33877-8.

## Introduction

Microalgae are a versatile group of microorganisms widely utilized as a source of biodiesel. They are also exploited for their main biochemical constituents such as pigments, carbohydrates, and proteins. Microalgae with high lipid productivity are suitable for biodiesel production, while those with high carbohydrate productivity could be fermented to produce other biofuels, such as bioethanol and biohydrogen (Gomaa et al. 2023). Biofuels are renewable energy sources that have been employed to overcome the worldwide energy problems associated with fossil fuels, which are mainly non-renewable. Promoting microalgae biomass and lipid productivities and facilitating their CO_2_ biofixation from the atmosphere are essential factors that allow for the economical production of biodiesel (Gomaa and Ali 2021; Gomaa et al. 2023).

The cultivation mode of microalgae directly influences microalgal biomass production and several cellular biochemical constituents. Microalgae exhibit different modes of nutrition, including photo-autotrophism, heterotrophism, and mixotrophism (Abreu et al. 2022). During photo-autotrophic growth, the cells can fix inorganic carbon (CO_2_ and bicarbonate) through light energy and photosynthesis. In contrast, under heterotrophic cultivation, the microalgal cells utilize organic compounds as both carbon and energy sources. Mixotrophy combines both photosynthesis and oxidation of organic carbons, taking advantage of both photo-autotrophic and heterotrophic mechanisms (Fawzy and Gomaa 2020). Autotrophy has the disadvantage of high CO_2_ supply costs and low biomass and lipid productivities. Heterotrophy or mixotrophy can promote lipid productivities compared to autotrophy, but the high cost of using organic carbon and susceptibility to microbial contamination are drawbacks. Generally, mixotrophic nutrition is preferred, as microalgal biomass productivity under mixotrophy exceeds that under autotrophy and heterotrophy (Abreu et al. 2022).

To reduce costs associated with mixotrophic cultivation, low-cost natural wastes have been used to promote microalgae growth and lipid productivity (Gomaa and Ali 2021). Additionally, cultivating microalgae under alkaline conditions can reduce microbial contamination (Chowdhury et al. 2019). For example, 2.0 × 10^−5^, 8.0 × 10^−5^, 7.0 × 10^−4^, and 7.0 × 10^−3^ mol CO_2_ per one liter of water are dissolved from the air (0.04 vol% CO_2_) at pH values of 6.0, 7.0, 8.0, and 9.0, respectively (Kikuchi et al. 2024). Consequently, alkaline conditions enhance CO_2_ scavenging from the atmosphere and CO_2_ biofixation rates, leading to higher microalgal growth (Vadlamani et al. 2017). However, most microalgae cannot thrive under alkaline conditions. For example, the freshwater microalgae *Chlamydomonas reinharditii* and *Auxenochlorella protothecoides* exhibited a marked reduction in cell growth at pH 8.5 and 9.0, respectively (Ochoa‐Alfaro et al. 2019; Andeden et al. 2021). As a result, one possible strategy for effectively sequestering CO_2_ from the atmosphere is to employ alkaliphilic microalgal strains. Additionally, an alkaline pH lengthens the time it takes for the cell cycle to complete and indirectly increases the generation of triacylglycerols (Andeden et al. 2021). Alkaliphilic algae, which thrive at high pH values (pH > 9), are considered promising candidates for large-scale growth and biofuel production (Vadlamani et al. 2017; Chowdhury et al. 2019).

Whey, a byproduct from dairy industries, causes serious environmental problems when discharged without proper treatment. The high lactose content in whey supports mixotrophic cultivation of several microalgae and cyanobacteria (Girard et al. 2014; Pereira et al. 2019; Abril Bonett et al. 2020). However, no attempts have been made to utilize cheese whey in the mixotrophic cultivation of alkaliphilic microalgae under different pH and NaNO_3_ concentrations. Additionally, there has been less research done on the effects of pH and NaNO_3_ on the lipid, protein, and carbohydrate in microalgae under mixotrophic feeding.

The present study aims to optimize the mixotrophic growth of two alkaliphilic microalgae (*Tetradesmus obliquus* and *Cyanothece* sp.) by varying cheese whey concentrations, culture pH, and NaNO_3_ levels. We investigate the effects of these factors on biomass productivity and the contents of main biochemical constituents (lipids, proteins, and carbohydrates). Furthermore, treatments with maximum lipid productivities are evaluated as potential biodiesel feedstock based on fatty acid composition and biodiesel characteristics.

## Materials and methods

### Microalgae and growth conditions

*Tetradesmus obliquus* and *Cyanothece* sp. (Fig. S1) were isolated from El-Ibrahimiya canal and River Nile at Assiut, Egypt, respectively. The microalgae were isolated, purified, and cultivated on a synthetic medium containing (g L^–1^): NaNO_3_, 2.5; K_2_HPO_4_, 0.5; K_2_SO_4_, 1.0; NaCl, 1.0; MgSO_4_·7H_2_O, 0.2; CaCL_2_·2H_2_O, 0.04; FeSO_4_·7H_2_O, 0.01; Na_2_-EDTA·2H_2_O, 0.08; H_3_BO_3_, 2.86 × 10^−3^; ZnSO_4_·7H_2_O, 2.22 × 10^−4^; NaMoO_4_·2H_2_O, 3.9 × 10^−4^; CuSO_4_·5H_2_O, 7.9 × 10^−5^, MnCl_2_·4H_2_O, 1.81 × 10^−3^; and Co(NO_3_)·6H_2_O, 4.94 × 10^−5^ in 1.0 L of distilled water. Algal cultivation occurred in 500-mL glass bottles under continuous illumination (48.4 μmol m^−2^s^−1^) at 25 ºC with air bubbling.

### Whey sampling and clarification

The whey (white cheese-whey from buffalo’s milk) was collected from a local dairy processing plant located et al.-Sharqia, Egypt. It was transported immediately to the laboratory in plastic bottles under refrigeration. The whey underwent heat treatment at 95 ºC for 90 min to clarify it. The precipitated material was removed by filtration (Pereira et al. 2019). The filtrate was collected and autoclaved separately before use to eliminate microbes and avoid growth of potential pathogens (Nazos et al. 2023). The concentrations of reducing sugars, total sugars, proteins, and lipids in the clarified cheese whey were estimated using 3,5-dinitrosalycylic acid method (Miller 1959), UV-H_2_SO_4_ method (Albalasmeh et al. 2013), Lowry method (Lowry et al. 1951), and phosphovanillin method (Mishra et al. 2014).

### Experimental design

The microalgal cells at exponential growth were inoculated into 200-mL sterilized synthetic medium in 500-mL glass bottles and supplemented with different concentrations of clarified whey. The synthetic medium was prepared as described in “Microalgae and growth conditions”. A central composite design (CCD) was applied to investigate the effects of whey concentration (0.5–4.5% (v/v)), initial pH (7–11), and NaNO_3_ concentration (0–2 g L^–1^) on the biomass productivity and cellular components. The three independent factors were investigated at five levels each and a total of 20 experiments including six replicates at the center point. The initial pH of the medium was adjusted prior to autoclaving. Microalgal cells’ initial concentration was set to 0.1 based on optical density at 750 nm. Cultures were incubated at 25 ± 2 °C, under continuous illumination (48.4 μmol m^−2^ s^−1^) and air bubbling (0.5 vvm) for 10 days. The following generalized form of a second-order polynomial equation was fitted to the experimental data:

1 $$Y={\beta }_{0}+\sum {\beta }_{i}{X}_{i}+\sum {\beta }_{ii}{X}_{i}^{2}+\sum {\beta }_{ij}{X}_{i}{X}_{j}$$

where the factors under investigation are *X*_*i*_ and *X*_*j*_, *Y* represents the expected response, and the regression, linear, quadratic, and interaction coefficients are, respectively, *β*_*o*_, *β*_*i*_, *β*_*ii*_, and *β*_*ij*_.

### Estimation of the microalgal growth and biomass productivity

A spectrophotometer (JENWAY 7315 Vis) was used to measure the optical density (OD) of the cultures at 750 nm in order to assess the microalgal growth. With the help of a standard curve (OD vs. dry cell weight, g L^−1^) unique to each alga, the OD data were converted into dry biomass weight. Thus, series of microalgal cultures were collected using centrifugation (4000 g, 15 min) with varying optical densities. The pellet was then oven-dried at 60 ºC to yield the corresponding dry cell weights. Using the following formula, the biomass productivity (BP, mg L^−1^ day^−1^) was determined (Gomaa et al. 2023):

2 $$BP=\frac{({X}_{t}-{X}_{0})}{\Delta t}$$

where *X*_*t*_ is the algal biomass at the end of experiment (mg L^−1^). *X*_0_ is the initial biomass concentration (mg L^−1^), and Δ*t* is the total duration of batch cultivation (day).

### Determination of lipids

Centrifugation was used to concentrate the microalgal cells, and they were then resuspended in a predetermined amount of distilled water. After adding 2 mL of concentric H_2_SO_4_ to 200 µL of concentrated microalgal cells, the mixture was heated for 10 min at 100 ºC in a water bath. Then, 5 mL of the phosphovanillin reagent was added to each tube (Mishra et al. 2014; Gomaa and Ali 2021). The absorbance was measured spectrophotometrically at 530 nm after 15 min. As a standard, sunflower oil was utilized.

### Determination of total proteins

According to Fawzy and Gomaa (2020), total proteins were extracted using 2 mL NaOH (1M) from 1 mL of concentrated cells at 100 ºC for 2 h. Following centrifugation, the supernatant was used to measure total proteins using the Lowry method (Lowry et al. 1951), with bovine serum albumin as the standard.

### Determination of soluble sugars

Distilled water was used to extract the soluble carbohydrates from the concentrated algal cells at 100 °C, 2 h. Using glucose as a standard, the phenol–sulfuric acid method was employed to quantify the soluble sugars in the supernatant following centrifugation (DuBois et al. 1956).

### Determination of FAME

The total lipids from the investigated microalgae under optimum growth conditions were extracted using chloroform/methanol (2:1) and converted into fatty acid methyl esters (FAME) as described previously (Johnson and Wen 2009). The FAME profile was identified using gas chromatography/mass spectrophotometry (GC/MS) in the Analytical Chemistry Unit, Faculty of Science, Assiut University, Egypt using the method reported previously (Fawzy et al. 2022).

### Biodiesel characteristics

The *FAME* profile of the *T. obliquus* and *Cyanothece* sp. was used for the estimation of several biodiesel characteristics using the following equations (Sarin et al. 2009; Ramírez-Verduzco et al. 2012):

3 $$\text{Saponification value}, SV= \sum (560\times N)/MW$$

4 $$\text{Iodine value}, IV=\sum (254\times N\times D)/MW$$

5 $$\text{Cetane number}, CN=46.3+5458/SV-(0.225\times IV)$$

6 $$\text{Degree of unsaturation}, DU=\sum MUFA+(2\times PUFA)$$

7 $$\text{Oxidation stability}, OS=-0.0384\times DU+7.77$$

8 $$\text{Long}-\text{chain saturation factor}, LCSF=\left(0.1\times C16:0\right)+\left(0.5\times C18:0\right)+\left(1\times C20:0\right)+\left(2\times C24:0\right)$$

9 $$\text{Cold filter plugging point}, CFPP=\left(3.1417\times LCSF\right)-16.477$$

10 $$\text{Cloud point}, CP=\left(0.526\times C16\right)-4.992$$

11 $$\text{Pour point}, PP=\left(0.571\times C16\right)-12.24$$

12 $$\text{Kinematic viscosity},\text{ ln}{v}_{i}=-12.503+(2.496\times \text{ln}MW)-(0.178 \times N)$$

13 $$\text{Density}, {\rho }_{i}=0.8463+(4.9/MW)+(0.0118\times N)$$

14 $$\text{Higher heating value}, HHV=46.19-(1794/MW)-(0.21\times N)$$

15 $$\text{Flash point}, FP=205.226+0.083\times C16:0-1.723\times C18:0-0.5717\times C18:1-0.3557\times C18:2-0.46\times C18:3-0.2287\times C22$$

where *MW* stands for molecular weight, *D* for the number of double bonds, *N* for the percentage of FAME, MUFA for monounsaturated fatty acids, and PUFA for polyunsaturated fatty acids.

### Statistical analysis

CCD analyses were performed using Chemoface v 1.65 (Nunes et al. 2012). The Pareto chart and response surface plots were generated using the same statistical program.

## Results

### Characterization of whey

The cheese whey sample contained reducing sugars, mainly lactose (6.67 g L^–1^), lipids (0.07 g L^–1^), protein (1.56 g L^–1^), and total carbohydrates (35.12 g L^–1^) (Table S1).

### Central composite design

The mixotrophic cultivation of the investigated microalgae using cheese whey at different pH and NaNO_3_ concentrations was optimized using central composite design (CCD), and the results are listed in Tables 1 and 2. For each alga, four second-order polynomial equations were obtained by the CCD to describe the relationship between the investigated variables and the responses (Table S2).

**Table 1:** Central composite design with uncoded factors (whey concentration (% v/v), pH, and NaNO_3_ (g L^−1^)) and the results of the investigated responses of *T. obliquus* (biomass productivity (BP, mg L^−1^ day^−1^), lipid content (L%), lipid productivity (LP, mg L^−1^ day^−1^), carbohydrate content (C%), carbohydrate productivity (CP, mg L^−1^ day^−1^), protein content (P%), protein productivity (PP, mg L^−1^ day^−1^)

No	Factors			Responses						
	Whey	pH	NaNO_3_	BP	L%	LP	C%	CP	P%	PP
1	1.5	8	0.5	30.22	20.07	6.06	10.22	3.09	20.64	6.24
2	1.5	8	1.5	48.02	28.71	13.78	10.35	4.97	20.24	9.72
3	1.5	10	0.5	36.91	32.03	11.88	8.05	2.97	18.99	7.02
4	1.5	10	1.5	40.51	26.08	10.56	10.15	4.11	19.55	7.92
5	3.5	8	0.5	31.61	33.35	10.53	17.77	5.60	23.34	7.38
6	3.5	8	1.5	32.11	28.69	9.21	10.61	3.40	26.51	8.51
7	3.5	10	0.5	48.69	42.39	20.64	14.45	7.02	22.53	10.97
8	3.5	10	1.5	45.00	22.84	10.31	7.58	3.43	19.65	8.83
9	1	9	1	47.44	23.55	11.16	10.74	5.10	19.54	9.27
10	4.5	9	1	48.09	33.80	16.25	13.58	6.53	28.49	13.70
11	2.5	7	1	26.39	18.74	4.93	15.94	4.21	23.10	6.10
12	2.5	11	1	34.16	22.62	7.71	10.17	3.47	19.67	6.69
13	2.5	9	0	32.78	39.95	13.09	13.34	4.37	13.90	4.55
14	2.5	9	2	45.50	25.75	11.74	8.78	4.00	16.68	7.59
15^a^	2.5	9	1	44.21	28.02	12.37	17.29	7.65	20.59	9.09

^a^Mean value of 5-replicates at the center point

**Table 2:** Central composite design with uncoded factors (whey concentration (% v/v), pH, and NaNO_3_ (g L^−1^)) and the results of the investigated responses of *Cyanotheca* (biomass productivity (BP, mg L^−1^ day^−1^), lipid content (L%), lipid productivity (LP, mg L^−1^ day^−1^), carbohydrate content (C%), carbohydrate productivity (CP, mg L^−1^ day^−1^), protein content (P%), protein productivity (PP, mg L^−1^ day^−1^)

No	Factors			Responses						
	Whey	pH	NaNO_3_	BP	L%	LP	C%	CP	P%	PP
1	1.5	8	0.5	8.20	16.18	1.33	10.38	0.85	20.22	1.66
2	1.5	8	1.5	37.01	9.97	3.69	17.79	6.58	19.38	7.17
3	1.5	10	0.5	30.07	8.63	2.59	7.55	2.27	13.60	4.09
4	1.5	10	1.5	25.84	7.95	1.95	12.96	3.35	19.45	4.96
5	3.5	8	0.5	34.63	18.91	6.56	5.11	1.77	16.41	5.68
6	3.5	8	1.5	45.67	18.47	8.43	14.99	6.84	16.77	7.66
7	3.5	10	0.5	41.14	11.83	4.88	6.65	2.78	8.85	3.68
8	3.5	10	1.5	34.23	9.50	3.27	10.34	3.54	16.02	5.49
9	1	9	1	22.77	13.96	3.18	13.75	3.13	22.04	5.02
10	4.5	9	1	52.78	21.65	11.42	8.14	4.31	14.95	7.89
11	2.5	7	1	32.63	20.65	6.74	7.18	2.33	15.51	5.05
12	2.5	11	1	29.25	8.54	2.53	9.94	2.88	12.25	3.56
13	2.5	9	0	0.00	0.00	0.00	0.00	0.00	0.00	0.00
14	2.5	9	2	21.11	3.17	0.67	20.60	4.35	17.00	3.59
15^a^	2.5	9	1	49.21	5.33	2.62	16.43	8.10	17.82	8.77

^a^Mean value of 5-replicates at the center point

All the equations exhibited high coefficient of determination (*R*^2^) values (*R*^2^ > 0.85, Table S2), implying the accuracy of the applied models. These results indicated that most of the variability in the responses could be attributed to the investigated variables.

### Effect of the investigated factors on biomass productivity

**Fig. 1:**
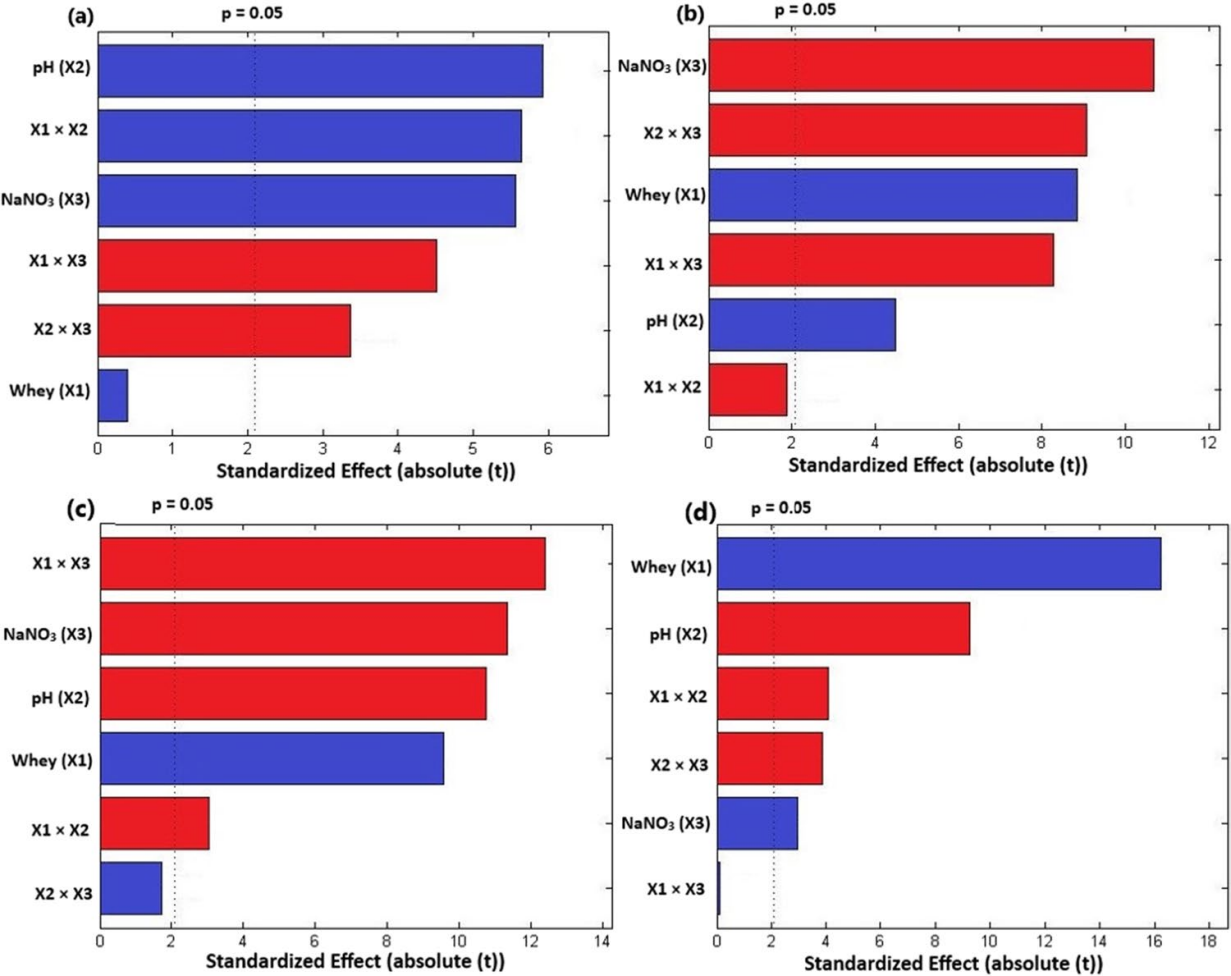
Pareto charts showing the effects of the investigated factors on **a** biomass productivity, **b** lipid content, **c** soluble carbohydrate content, and **d** total protein content of *Tetradesmus obliquus*. Blue bars indicate positive effects and red bars indicate negative effects

**Fig. 2:**
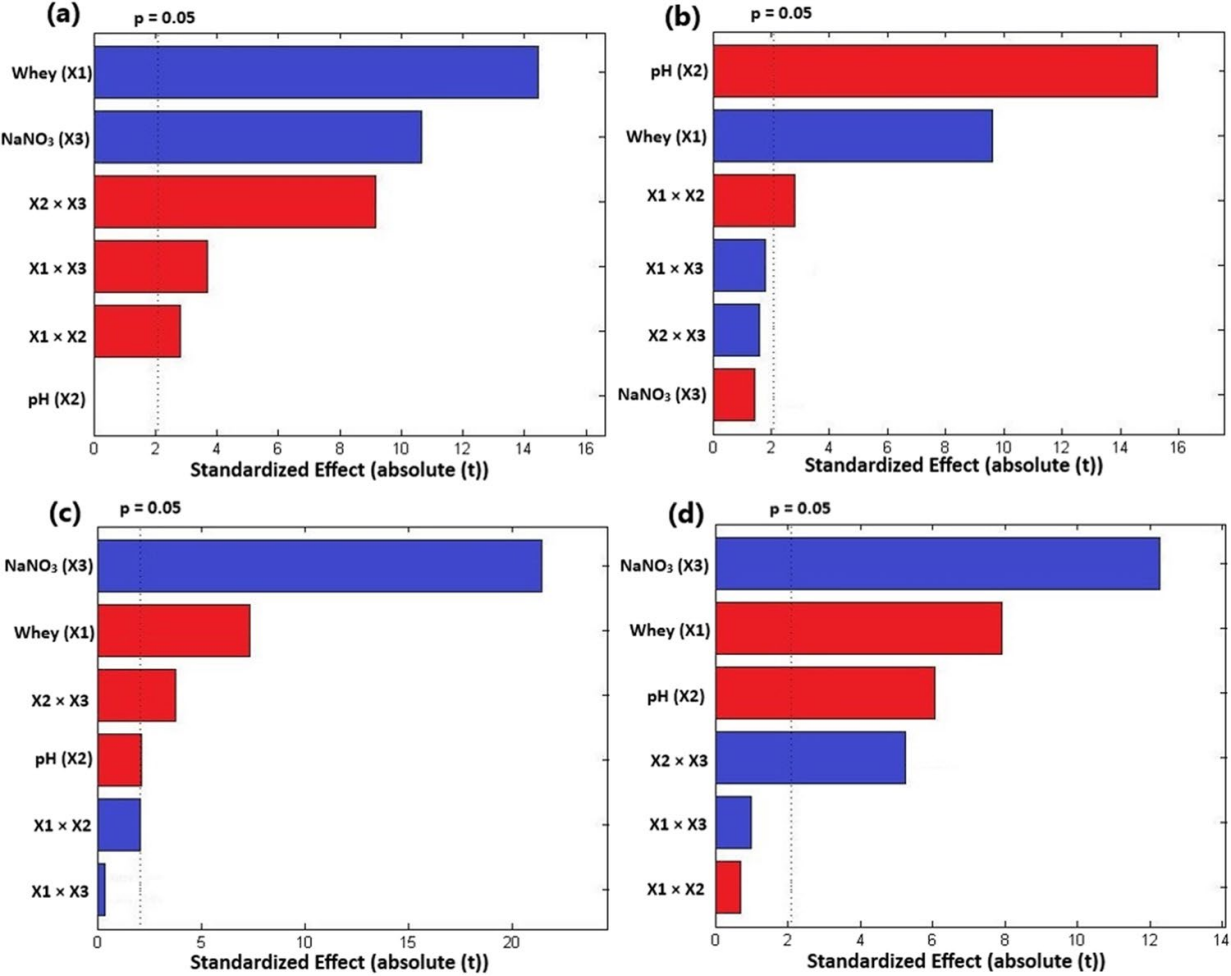
Pareto charts showing the effects of the investigated factors on **a** biomass productivity, **b** lipid content, **c** soluble carbohydrate content, and **d** total protein content of *Cyanothece*. Blue bars indicate positive effects and red bars indicate negative effects

The Pareto chart was applied to detect the significance of the investigated factors and their effects (positive or negative). As depicted in Fig. 1a, pH and NaNO_3_ significantly influenced (*p* < 0.05) the biomass productivity (BP) of *T. obliquus*, while for *Cyanothece*, whey and NaNO_3_ concentrations played a significant role (Fig. 2a). Furthermore, the mutual interaction between the three investigated factors also had significant effects on the BP of both microalgae (Figs. 1a and 2a). Specifically, pH and the interaction between whey concentration and pH had the greatest impact on the BP of *T. obliquus*, whereas in the case of *Cyanothece*, the highest effects were related to whey and NaNO_3_ concentration (Figs. 1a and 2a).

**Fig. 3:**
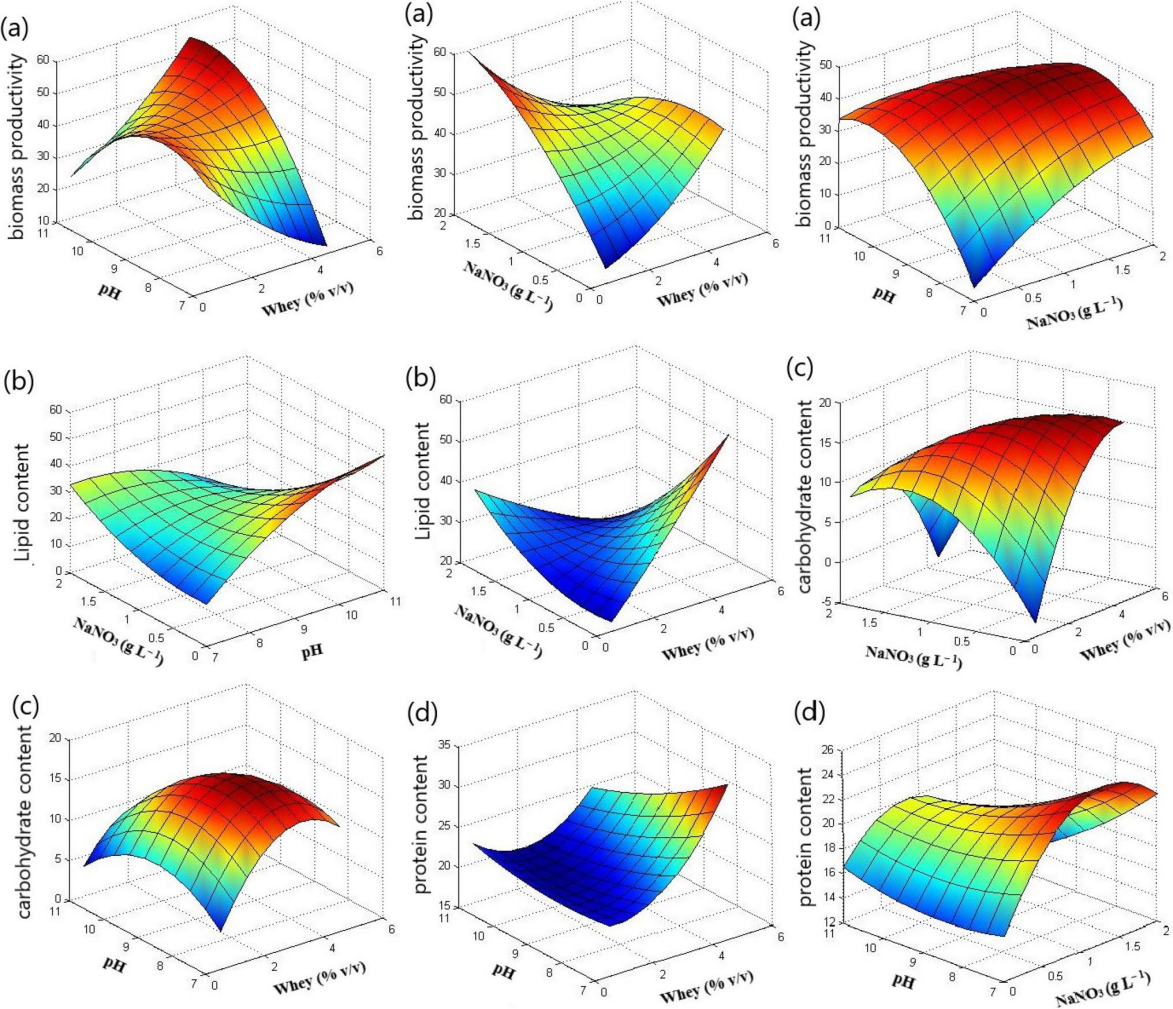
3D response surface plots showing the effects of the investigated factors and their interactions on **a** biomass productivity, mg L^−1^ day^−1^, **b** lipid content (% w/w), **c** soluble carbohydrate content (% w/w), and **d** total protein content (% w/w) of *Tetradesmus obliquus*

The BP of *T. obliquus* fluctuated between 26.39 and 48.69 mg L^–1^ day^–1^ (Table 1). The BP of this microalga exhibited non-significant variations under the investigated whey concentrations. However, increasing the initial pH of the culture medium of *T. obliquus* markedly enhanced its BP, especially at higher whey concentrations (Figs. 1a and 3a). Therefore, pH and its mutual interaction with whey concentration showed positive effects on the BP of *T. obliquus* (Fig. 3a). Similarly, NaNO_3_ concentration exhibited positive effects on the BP of *T. obliquus*, but its mutual interactions with whey concentration and pH were negative (Fig. 1a and 3a).

**Fig. 4:**
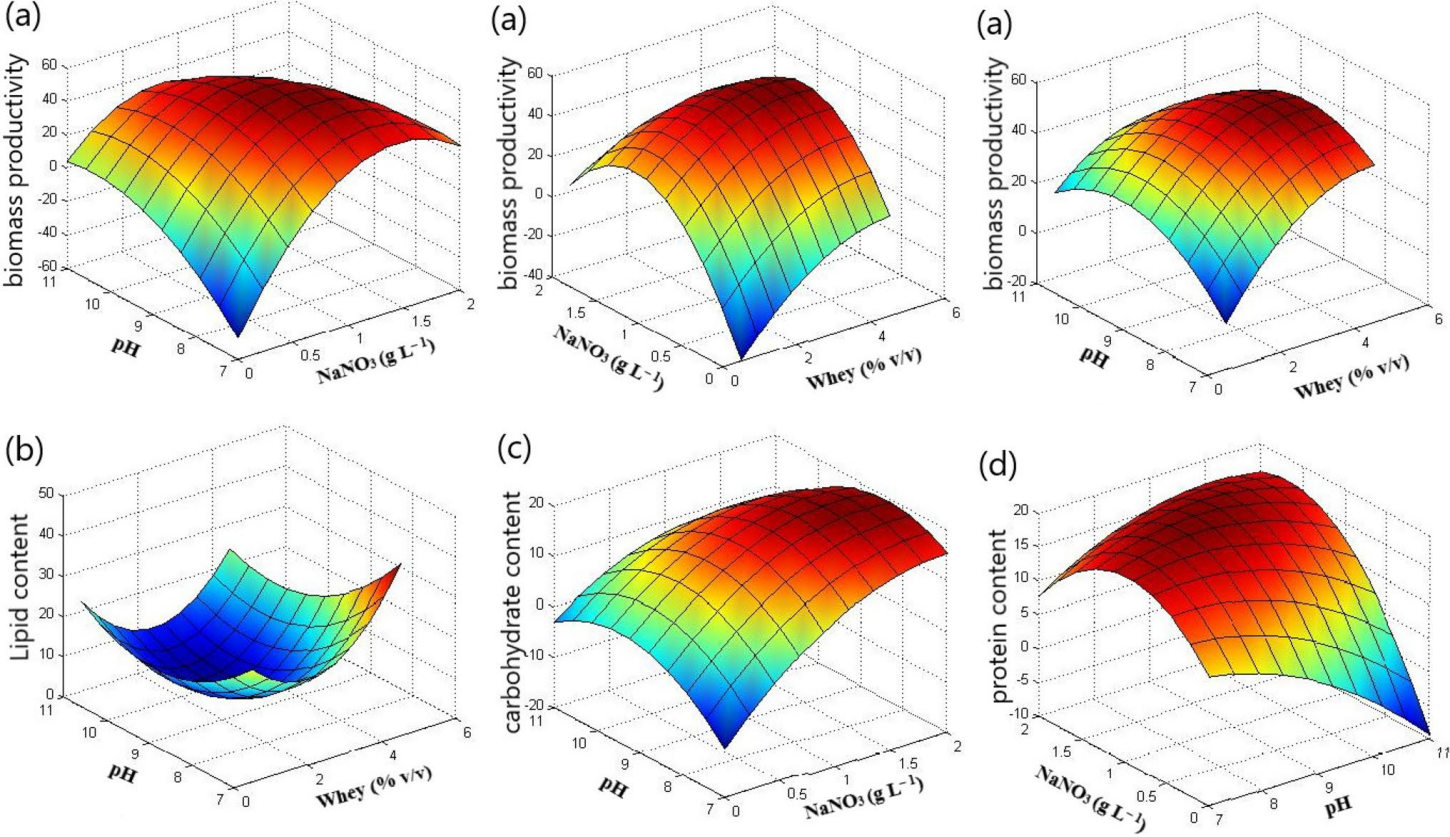
3D response surface plots showing the effects of the investigated factors and their interactions on **a** biomass productivity, mg L^−1^ day^−1^, **b** lipid content (% w/w), **c** soluble carbohydrate content (% w/w), and **d** total protein content (% w/w) of *Cyanothece*

On the other hand, the cyanobacterium *Cyanothece* failed to grow under nitrogen-deprived conditions, resulting in a drastic decline in growth. Consequently, its BP showed negative values at 0.0 g L^–1^ of NaNO_3_ (Table 2; Fig. 4a). However, in the presence of nitrate at different culture conditions, the BP of *Cyanothece* ranged from 8.20 to 52.78 mg L^–1^ day^–1^ (Table 2). Increasing the initial concentration of NaNO_3_ exhibited positive effects on its BP (Figs. 2a and 4a). Similarly, increasing whey concentration positively influenced the cyanobacterium’s growth. Nevertheless, the three investigated factors exhibited antagonistic mutual interactions on the BP of *Cyanothece* (Figs. 2a and 4a).

### Effect of the investigated factors on lipid contents

The lipid content (LC) of *T. obliquus* fluctuated between 18.74% w/w (run 11) and 42.39% w/w (run 7), corresponding to lipid productivity of 4.93 and 20.64 mg L^–1^ day^–1^, respectively (Table 1). Meanwhile, the LC within *Cyanothece* cells ranged from 3.17% w/w (run 14) to 21.65% w/w (run 10), with corresponding lipid productivity of 0.67 and 11.42 mg L^–1^ day^–1^, respectively (Table 2).

The three investigated factors significantly affected the LC of *T. obliquus* (Fig. 1b). In contrast, the LC of *Cyanothece* was notably influenced by pH and whey concentration (Fig. 2b). The most prominent effects on the LC of *T. obliquus* and *Cyanothece* were related to nitrogen concentration and pH, respectively (Figs. 1b and 2b). However, the LC exhibited a drastic decrease with increasing the initial concentration of NaNO_3_ in *T. obliquus* culture, especially at higher whey concentration and pH (Fig. 3b). Consequently, negative effects on LC of *T. obliquus* were observed for NaNO_3_ concentration and its mutual interaction with whey and pH (Fig. 1b). Additionally, initial culture pH exhibited significant positive effects on the LC content of *T. obliquus* at low nitrate concentration (Fig. 1b). The interaction between initial culture pH and nitrate concentration was also negative (Fig. 3b).

On the other side, increasing the initial pH during *Cyanothece* cultivation adversely affected its LC (Fig. 2b). Conversely, the LC was significantly increased at higher whey concentrations, but antagonistic interactions between whey concentration and culture pH were observed (Figs. 2b and 4b).

### Effect of the investigated factors on SCC

The soluble carbohydrate contents (SCC) of* T. obliquus* ranged from 7.58 to 17.77% (w/w) (Table 1), while in *Cyanothece*, the SCC fell within the range 5.11–20.60% (w/w) (Table 2). The three investigated factors exhibited significant effects on SCC of both microalgae (Figs. 1c and 2c). Increasing NaNO_3_ concentration had prominent positive effects on SSC of *Cyanothece* (Fig. 2c), but negative effects in the case of *T. obliquus* (Fig. 1c)*.* Additionally, pH showed negative effects on SCC of both microalgae. However, increasing whey concentration in *T. obliquus* culture had positive effects on SCC, although its interaction with pH and nitrate resulted in significant negative effects (Figs. 1c and 3c). Notably, the mutual whey and nitrate interaction had the greatest impact on the SCC of *T. obliquus* (Figs. 1c and 3c)*.* Furthermore, pH significantly interacted antagonistically with NaNO_3_ concentration regarding the SSC of *Cyanothece* (Figs. 2c and 4c)*.*

### Effect of the investigated factors on TPC

The total protein contents (TPC) for *T. obliquus* and *Cyanothece*, as assessed by the Pareto diagram, indicated that the three variables under consideration were statistically significant (*p* < 0.05) (Figs. 1d and 2d). The TPC of *T. obliquus* ranged from 13.90 to 28.49% (w/w), while for *Cyanothece* sp., it fell within the range of 8.85 to 22.04% (w/w) (Tables 1 and 2, respectively).

In *T. obliquus*, whey concentration exerted the greatest influence on TPC, whereas in the case of *Cyanothece*, the highest effects were associated with the initial NaNO_3_ (Figs. 1d and 2d). Interestingly, increasing whey concentration had a positive effect on the TPC in *T. obliquus* (positive effects) (Fig. 1d), but the opposite trend was observed in *Cyanothece* (Fig. 2d). Both microalgae exhibited increased TPC at nitrogen replete conditions, with NaNO_3_ showing positive linear effects on TPC (Figs. 1d and 2d).

However, higher pH values in the culture medium were not conducive to cellular protein accumulation in either *T. obliquus* or *Cyanothece* (Figs. 1d and 2d). Notably, these negative effects of pH were exacerbated at higher NaNO_3_ concentrations in *T. obliquus* (Fig. 3d) and at low NaNO_3_ concentrations in *Cyanothece* (Fig. 4d). Consequently, the mutual interaction between pH and NaNO_3_ regarding TPC was antagonistic in *T. obliquus* (Fig. 1d) but synergistic in *Cyanothece* (Fig. 2d). Additionally, a negative mutual interaction between pH and whey was observed in *T. obliquus* (Fig. 1d).

### FAME composition and biodiesel properties

The treatments with the highest lipid productivity (treatment no. 7 for *T. obliquus* and treatment no. 10 for *Cyanothece* sp*.*) were analyzed to identify the fatty acid methyl esters (FAME) and their corresponding biodiesel characteristics. The FAME profile indicated higher percentages of saturated fatty acids (SFAs), which contributed to 56.96% and 79.38% of the total percentage of fatty acids (FAs) in *T. obliquus* and *Cyanothece* sp*.*, respectively (Table S3). The remaining percentage was related to monounsaturated fatty acids (MUFAs), and no polyunsaturated fatty acids (PUFAs) were detected for both microalgae.

In *T. obliquus*, the percentage of palmitic acid reached 53.92%, followed by oleic acid (18.08%) and cis-vaccenic acid (14.56%). Meanwhile, in *Cyanothece* sp*.*, the percentage of palmitic acid was 65.98%, followed by n-capric acid (13.41%), oleic acid (10.31%), and cis-vaccenic acid (10.31%) (Table S3).

Based on the FAME profile, several biodiesel characteristics were calculated, and the results are listed in Table 3. The saponification values (SV), representing the amount of potassium hydroxide required to saponify 1 g of oil, were 208.03 mg KOH g^−1^ fat for *T. obliquus* and 228.53 mg KOH g^−1^ fat for *Cyanothece*. Notably, the iodine values for both microalgae were low and significantly lower than the limit specified in the European standard (EN 14214). Similarly, the cetane number (CN) values for both microalgae exceeded the limits set by EN 14214, American standard (ASTM D6751), and Indian standard (IS 15607).

**Table 3:** Biodiesel characteristics of *T. obliquus* and *Cyanothece* sp. cultivated under the optimized mixotrophic conditions compared with international biodiesel standards and biodiesel properties of other microalgae cultivated under mixotrophic cheese whey conditions

Species	SV (mg KOH g^−1^ fat)	IV (g *I*_2_ 100 g^−1^ fat)	CN	DU (% wt)	OS (h)	LCSF (% wt)	CFPP (°C)	CP (°C)	PP (°C)	*υ* (mm^2^ s^−1^)	*ρ* (g cm^−3^)	HHV (MJ kg^−1^)	FP (°C)
*Tetradesmus obliquus*	208.03	38.61	63.85	43.04	6.12	5.39	0.46	23.36	18.55	4.8	0.87	40.11	186.29
*Cyanothece* sp.	228.53	18.54	66.01	10.31	7.37	6.6	4.25	29.71	25.43	3.11	0.88	39.41	204.81
*Desmodesmus* sp.^a^	200.74	77.12	56.14	79.49	8.45	6.28	3.25	8.63	2.54	3.69	0.85	38.44	ND
*Leptolyngbya*-rich consortium^b^	200.08	91.47	52.6	ND	7.37	3.68	− 3.94	6.62	ND	3.29	0.86	37.26	ND
*Chlamydomonas* sp.^c^	220.85	102.32	47.99	76.08	4.38	0.35	− 11.03	ND	ND	4.31	ND	41.70	ND
*Chlorella* sp.^c^	221.76	54.89	58.56	41.26	3.56	1.73	− 15.37	ND	ND	3.95	ND	39.50	ND
*Chlorella vulgaris*^d^	ND	12.42	54.88	ND	ND	ND	ND	− 9.42	8.57	4.82	0.86	42.97	115
*T. obliquus*^e^	207.82	27.95	66.27	28.56	6.67	14.84	31.93	22.78	17.92	3.62	0.88	40.06	165.76
EN 14214	-	≤ 120	≥ 51	-	≥ 6	-	≤ 5/ − 20	-	-	3.5–5.0	0.86–0.90	-	120
ASTM D6751-02	-	-	≥ 47	-	≥ 6	-		-	-	1.9–6.0	0.86–0.90	-	93
IS 15607	-	-	≥ 51	-	≥ 6	-	18 Jun	-	15 Mar	2.5–6.0	0.86–0.90	-	-

*EN* European Committee for Standardization, *ASTM* American Society for Testing and Materials, *IS* Indian standard, *SV* saponification value, *IV* iodine value, *CN* cetane number, *DU* degree of unsaturation, *OS* oxidation stability, *LCSF* long-chain saturation factor, *CFPP* cold filter plugging point, *CP* cloud point, *PP* pour point, *υ* kinematic viscosity, *ρ* density, *HHV* higher heating value, *FP* flash point

^a^Cultivated in 50% BBM and 15% cheese whey (Salah et al. 2023)

^b^Cultivated in 8% cheese whey (Tsolcha et al. 2018)

^c^Cultivated in BG-11 medium containing 40% cheese whey (Mondal et al. 2021)

^d^Cultivated in Jaworski’s medium and cheese whey (1:11 v/v) (Choi 2016)

^e^Cultivated in Bold’s Basal medium containing 4 g L^−1^ NaHCO_3_ (Fawzy et al. 2022)

In terms of unsaturation, the degree of unsaturation (DU) in *Cyanothece* biodiesel reached 10.31 wt.%, contrasting with 43.04 wt.% in *T. obliquus*. The oxidation stability (OS) values for both microalgae complied with international standards, exceeding a value of 6. Additionally, the cold filter plugging point (CFPP) estimates were 0.46 °C and 4.25 °C, aligning with the limits specified by the EN 14214 standard and IS 15607. Both microalgae exhibited cloud point (CP) and pour point (PP) values above 18 °C.

Furthermore, the kinematic viscosity (*υ*) and density (*ρ*) fell within the limits specified by international standards. Lastly, the higher heating values (HHV) were 39.41 MJ kg^−1^ for *T. obliquus* and 40.11 MJ kg^−1^ for *Cyanothece* sp*.*

## Discussion

The growth behavior and accumulation of value-added metabolites in two microalgae were investigated at varying organic carbon, nitrogen, and pH concentrations. Importantly, increasing culture pH enhances CO_2_ uptake rates from the atmosphere and provides a higher supply of bicarbonate as well as it reduces culture crashes due to contamination or the establishment of predators (Vadlamani et al. 2017). The microalga is considered alkalitolerant when it prefers to grow under neutral conditions, with significantly less growth observed at alkaline pH. In contrast, alkaliphilic microalgae thrive optimally at pH 9–10 (Gimmler and Degenhard 2001). Notably, the significant positive effects of pH on the biomass productivity of *T. obliquus* indicated its alkaliphilic nature, with optimal growth at pH 9–10. In contrast, the growth of the cyanobacterium *Cyanothece* sp. was not inhibited at alkaline pH, further confirming its alkaliphilic characteristics. However, its biomass production exhibited insignificant effects upon increasing culture pH. Alkaliphilic microalgae and cyanobacteria can efficiently utilize bicarbonate as a carbon source, facilitated by the action of carbonic anhydrase. The higher CO_2_ biofixation rates and increased biomass productivity observed in alkaliphilic microalgae compared to neutralophilic species offer economic and environmental advantages.

During mixotrophic cultivation, both investigated microalgae effectively utilized cheese whey as a source of organic carbon, aligning with previous studies (Girard et al. 2014; Pereira et al. 2019; Tharani and Ananthasubramanian 2020; Bentahar and Deschênes 2022). In this nutritional mode, microalgal cells benefit from both inorganic and organic carbon sources, promoting simultaneous growth and the production of certain metabolites (Gomaa and Yousef 2020; Gomaa and Ali 2021).

The growth of microalgae on cheese whey necessitates the biosynthesis of β-galactosidase, which enables the conversion of lactose in the whey into glucose and galactose. This mechanism has been previously confirmed in various microalgae, including *T. obliquus* and *Cyanothece* sp. (Suwal et al. 2019; Zanette et al. 2019; Chenebault et al. 2020; Bentahar and Deschênes 2022). A detailed comparison of the growth and biochemical composition of the investigated microalgae in relation to previous studies regarding mixotrophic cultivation using cheese whey is listed in Table S4. In the present study, the mixotrophic cultivation was performed under alkaline conditions using low concentration of cheese whey. This is advantageous for large-scale production to minimize microbial contamination and culture crashing. The biomass and lipid productivity of the investigated microalgae were comparatively higher than those reported for *Chlorella vulgaris* (Salati et al. 2017; Melo et al. 2018), *Desmodesmus* sp. (Salah et al. 2023), *Chlamydomonas* sp. (Mondal et al. 2021), and *Spirulina platensis* (Pereira et al. 2019).

The results of the current study indicated that, at low cheese whey concentrations, the supply of bicarbonate through an increase in culture pH becomes more critical for sustaining *T. obliquus* growth than higher whey concentrations. Specifically, raising the culture pH from 8 to 10 at 1.5% (v/v) whey and 0.5 gL^−1^ NaNO_3_ led to an enhancement of biomass productivity of *T. obliquus* from 30.22 to 36.91 mg L^−1^ day^−1^. Interestingly, at slightly alkaline pH conditions (pH 8), increasing the cheese whey concentration to 3.5% (v/v) did not significantly affect the biomass productivity of *T. obliquus* (31.61 mg L^−1^ day^−1^)*.* These effects may be related to the variation of β-galactosidase production and activity under different culture conditions. For example, Bentahar et al. reported that increasing lactose concentration in *T. obliquus* culture did not elevate the specific activity of β-galactosidase (Bentahar et al. 2019). However, the culture pH plays a crucial role, as β-galactosidase activity is enhanced at alkaline pH in microalgae (Zanette et al. 2019).

The present results indicated that cheese whey concentration had non-significant linear effects on the biomass productivity of *T. obliquus*, while culture pH exhibited remarkable positive effects. However, synergistic significant effects of whey concentration and culture pH were observed. This implies that better biomass production of *T. obliquus* occurs at high whey concentration and pH, utilizing both inorganic and organic carbon. Consequently, the highest biomass productivity of 48.69 mg L^−1^ day^−1^ was observed at 3.5% (v/v) of whey, pH 10, and 0.5 g L^−1^ NaNO_3_. This value was approximately 1.6–1.8 times higher than those observed at pH 7 and 8.

Similarly, Zhang et al. reported optimal growth of *Scenedesmus obliquus* at pH 10 under autotrophic conditions (Zhang et al. 2019). In another study, mixotrophic cultivation of *S. obliquus* using disperse orange‑2RL Azo dye induced high biomass production under alkaline conditions (pH 11) (Hamouda et al. 2022). The shift of culture pH toward alkalinity can lead to different gene expression in the microalgal cells compared to neutral pH. For instance, 1432 genes in alkali-tolerant *Chlorella* sp. BLD were relatively upregulated at pH 10 compared to pH 7.5 (Qu and Miao 2021). The upregulation of genes related to CO_2_ fixation and photosynthesis ensures higher algal growth under alkaline conditions (Qu and Miao 2021).

On the other hand, the growth variation of *Cyanothece* sp. was primarily influenced by the supply of organic carbon through cheese whey, rather than variations in pH. Consequently, the maximum biomass productivity of *Cyanothece* sp. reached 52.78 mg L^−1^ day^−1^ at 4.5% (v/v) whey, pH 9, and 1.0 gL^−1^ NaNO_3_. Remarkably, this value was 2.3-fold higher than that obtained at low whey concentration (1.0% v/v) under the same conditions. These observations suggest that the assimilation of cheese whey by *Cyanothece* sp. as a carbon source is more effective for biomass production, in contrast to the results observed for *T. obliquus.* These variations may be related to the variation in β-galactosidase production and activity in microalgae (Zanette et al. 2019).

During mixotrophic cultivation, the availability of sufficient carbon not only influences microalgae growth but also impacts their biochemical composition (Gomaa and Ali 2021). Multiple correlation analysis revealed that treatments with higher biomass production of *T. obliquus* exhibited significant correlations with increased cellular lipid content (*R* = 0.493, *p* = 0.027). This relationship is advantageous for ensuring higher lipid productivity for biodiesel synthesis. However, non-significant correlations (*p* > 0.05) were observed between lipid, protein, and carbohydrate in the case of *T. obliquus*. These observations suggest that incorporating cheese whey into *T. obliquus* culture provided sufficient carbon for both cellular division and the synthesis of lipids, proteins, and carbohydrates. Consequently, our results indicated a significant increase in lipid, protein, and carbohydrate contents with increasing cheese whey concentration during mixotrophic cultivation.

In contrast, the lipid content of *Cyanothece* sp. was not directly related to its biomass production but showed an inverse relationship with carbohydrate content (*R* =  − 0.572, *p* = 0.01). Furthermore, protein and carbohydrate contents in *Cyanothece* sp. exhibited a positive correlation (*R* = 0.542, *p* = 0.017). These observations suggest that *Cyanothece* cells effectively utilized cheese whey as a carbon source, allocating it primarily to lipid synthesis. Consequently, our results indicated a significant increase in lipid content in *Cyanothece* cells, accompanied by a decrease in protein and carbohydrate contents with increasing whey concentration in the culture medium. Interestingly, the growth of *Spirulina platensis* on cheese whey led to the accumulation of protein content at the expense of reduced carbohydrate synthesis (Pereira et al. 2019).

Microalgal metabolism is greatly influenced, either directly or indirectly, by the culture pH. Increasing the culture pH led to higher lipid accumulation in the case of *T. obliquus*, but it lowered the lipid content in *Cyanothece* cells. Previous studies have also observed triacylglyceride accumulation in *T. obliquus* cells when culture pH was increased, especially under nitrogen-deprived conditions (Gardner et al. 2011; Breuer et al. 2013; Andeden et al. 2021).

In our current study, we observed a remarkable increase in lipid content in *T. obliquus* under high initial pH conditions and low NaNO_3_ concentrations. Specifically, the highest lipid content of 42.39% (w/w) was obtained at 3.5% (v/v) cheese whey, pH 10, and NaNO_3_ 0.5 g L^–1^. Under these conditions, lipid productivity reached 20.64 mg L^–1^ day^–1^. However, this value was nearly halved when the initial culture pH was set to 8.0 or NaNO_3_ concentration was increased to 1.5 g L^–1^. These results align with the effects of alkaline conditions on lipid synthesis in *Chlorella* sp. (Qu and Miao 2021).

The increase in lipid content in *T. obliquus* can be attributed to the elevation of acetyl-coenzyme A, a precursor for fatty acid (FA) biosynthesis. This elevation occurs due to pyruvate accumulation from CO_2_ fixation, as well as protein and carbohydrate hydrolysis (Qu and Miao 2021). Consequently, the decrease in soluble carbohydrates and total proteins resulting from increasing the initial culture pH in the investigated microalgae may be related to the upregulation of genes responsible for starch and protein hydrolysis, as reported previously (Qu and Miao 2021).

The decrease in lipid contents of *Cyanothece* cells at high pH agreed with previous observations on the cyanobacterium *Leptolyngbya foveolarum* (Singh and Kumar 2021). Unfortunately, *Cyanothece* sp. was characterized by low lipid accumulation compared to *T. obliquus*, which reached a maximum value of 21.65% (w/w) and 11.42 mg L^−1^ day^−1^ at 4.5% (v/v) cheese whey, pH 9.0, and NaNO_3_ 1.0 g L^−1^. However, the obtained results for lipid contents were relatively higher than those reported in several cyanobacterial species belonging to genera *Oscillatoria*, *Phormidium*, *Lyngbya*, *Leptolyngbya*, *Nostoc*, *Spirulina*, and *Synechococcus*, where the lipid contents fluctuated between 2.3% and 10.6% (Mathimani et al. 2018; Yadav et al. 2021).

Nitrogen concentration in the growth medium is a fundamental factor that affects microalgal growth and biosynthesis of cellular metabolites. Increasing NaNO_3_ concentrations supported the growth of both *T. obliquus* and *Cyanothece* sp. However, a complete inhibition of *Cyanothece* sp. growth was observed under nitrogen-deprived conditions, which contradicts the behavior of *T. obliquus*. Previous studies have reported similar trends: a decline in the growth of the cyanobacterium *Aphanocapsa* sp. under nitrogen starvation (Gomaa and Ali 2021) and nitrogen starvation-induced growth inhibition in the cyanobacterium *Synechocystis* sp. through the downregulation of genes associated with photosynthesis, protein biosynthesis, and energy metabolism (Huang et al. 2013). Under nitrogen-starved or limited conditions, several microalgae tend to hyperaccumulate lipids and increase carbohydrate synthesis while decreasing protein contents (Nagappan et al. 2020).

In the case of *T. obliquus*, the present results indicated a significant increase in lipid and carbohydrate contents, with a concomitant decrease in protein contents, by decreasing NaNO_3_ concentration in the medium. Conversely, increasing nitrate concentrations in the culture of *Cyanothece* sp. significantly increased carbohydrate and protein contents, but without significant effects on lipid contents. The optimum growth conditions for obtaining higher biomass, lipid, protein, and carbohydrate productivities from *T. obliquus* were 3.5% (v/v) cheese whey, pH 10.0, and 0.5 g L^−1^ NaNO_3_. Under these conditions, the biomass, lipid, protein, and carbohydrate productivities were 48.69, 20.64, 7.02, and 10.97 mg L^−1^ day^−1^, respectively. On the other hand, at 4.5% (v/v) cheese whey, pH 9.0, and 1.0 g L^−1^ NaNO_3_, *Cyanothece* exhibited a higher biomass of 52.78 mg L^−1^ day^−1^ and lipid productivities of 11.42 mg L^−1^ day^−1^. To increase carbohydrate and protein productivities from *Cyanothece* sp., the cheese whey concentration should be reduced to 2.5% (v/v) at the same pH and NaNO_3_ concentration. Consequently, the carbohydrate and protein productivities would increase to 8.10 and 8.77 mg L^−1^ day^−1^.

A detailed comparison of the main fatty acids in the investigated microalgae with other microalgae under mixotrophic cultivation is listed in Table S5. The biodiesel obtained from the investigated microalgae was characterized by a higher percentage of SFAs, followed by MUFAs, and no PUFAs were detected. Lipids with high contents of SFAs and MUFAs can produce high-quality biodiesel (Gomaa et al. 2023). The FAME profile for both microalgae was rich in C16 and C18 FAs (Table S5). Under mixotrophic conditions, lipids rich in SFAs and MUFAs were produced, but PUFAs were not detected in both microalgae. Consequently, the produced biodiesel exhibited low IV and DU, along with high OS, indicating stable biodiesel suitable for long-term storage (Gomaa and Ali 2021). Under the optimized conditions, the present microalgae produced biodiesel with lower IV and higher CN than those reported for other microalgae (Table 3).

The CN reflects the ignition efficiency of the engine, and the obtained values for both microalgae could ensure better engine performance. The biodiesel produced by *Cyanothece* sp. showed better CN, DU, IV, and OS compared to that of *T. obliquus* under the optimized conditions. The ν (kinematic viscosity) values were related to the flow performance of the biodiesel in the engine. Generally, *ν* increases with increasing long-chain FAs, DU, and trans-oriented FAs. Accordingly, the biodiesel from *T. obliquus* had higher *ν* values than *Cyanothece* sp., but both were within the limits specified by international standards.

The CP (cloud point), PP (pour point), and CFPP (cold filter plugging point) were indicative of biodiesel flow properties in cold climates. While PP and CP were not specified in international standards, the IS standard indicated a minimum of three and 15 for PP during winter and summer, respectively. The values obtained in the present study indicated better performance of *T. obliquus* biodiesel in cold climates compared to *Cyanothece* sp. Furthermore, the CFPP values for both microalgae agreed with international limits. The HHV (higher heating value) defines the amount of heat produced after combustion of 1 g of biodiesel. Although not identified by international standards, HHV usually ranges between 39 and 41 MJ kg^−1^ for microalgal-derived fuel (Demirbas 2009), which aligns with the findings of this study.

## Conclusion

The present study evaluated the mixotrophic growth and productivity of two alkaliphilic microalgae using cheese whey as a cheap, environmentally benign, and sustainable organic carbon source. We investigated algal biomass productivity and the contents of lipids, total proteins, and soluble carbohydrates under different concentrations of cheese whey, nitrate, and culture pH. Incorporating cheese whey into the culture medium of *T. obliquus* had positive effects on lipid, carbohydrate, and protein contents, enabling higher biomass productivity at elevated culture pH. Similarly, the culture of *Cyanothece* exhibited higher biomass productivity and lipid contents at high concentrations of cheese whey, albeit with a concomitant decrease in protein and soluble carbohydrate contents. Increasing culture pH supported higher biomass productivity with elevated cellular lipid contents in *T. obliquus*, but protein and carbohydrate levels decreased. Conversely, increasing culture pH during *Cyanothece* cultivation significantly decreased its lipid and protein contents, with no significant effects on biomass production. Nitrate deficiency increased lipid and soluble carbohydrate contents in *T. obliquus* during mixotrophic cultivation, but biomass productivity decreased simultaneously. Nitrate was crucial for supporting *Cyanothece* growth and increasing cellular protein and carbohydrate contents. Accordingly, the optimum conditions to promote biomass and lipid productivity in *T. obliquus* were 3.5% (v/v) cheese whey, initial pH 10.0, and 0.5 g L^−1^ NaNO_3_. Under these conditions, the biomass, lipid, soluble carbohydrate, and protein productivities were 48.69, 20.64, 7.02, and 10.97 mg L^−1^ day^−1^, respectively. Meanwhile, at 4.5% (v/v) cheese whey, initial pH 9.0, and 1.0 g L^−1^ NaNO_3_, *Cyanothece* exhibited biomass, lipid, soluble carbohydrate, and protein productivities of 52.78, 11.42, 4.31, and 7.89 mg L^−1^ day^−1^, respectively. Furthermore, the biodiesel produced under these conditions exhibited favorable characteristics that aligned with international standards. Overall, this study highlights the promising utilization of alkaliphilic microalgae in biofuel production through cost-effective mixotrophy.

### Supplementary Information

Below is the link to the electronic supplementary material.

Supplementary file1 (PDF 332 KB)

## Data Availability

The datasets used and/or analyzed during the current study are available from the corresponding author on reasonable request.
