# Supplementary material for: Enhancement of biomass productivity and biochemical composition of alkaliphilic microalgae by mixotrophic cultivation using cheese whey for biofuel production
Source: Environ Sci Pollut Res Int. 2024 Jun 17;31(30):42875–88. doi: 10.1007/s11356-024-33877-8 (PMC11222269; doi:10.1007/s11356-024-33877-8)
Supplement: Supplementary file 1 — Supplementary file1 (PDF 332 KB) [file 11356_2024_33877_MOESM1_ESM.pdf]

SUPPLEMENTRY MATERIAL

Enhancement of biomass productivity and biochemical composition of alkaliphilic microalgae by mixotrophic cultivation using cheese whey for biofuel production

Ahmed Mohamed Youssef[1], Mohamed Gomaa[2,*], Abdel Kareem S. H. Mohamed[1], Abdel-Rahim A. El-Shanawany[1]

[1] Department of Botany and Microbiology, Faculty of Science, Al-Azhar University, Assiut 71524, Egypt

[2] Botany & Microbiology Department, Faculty of Science, Assiut University, 71516, Assiut, Egypt

***Corresponding author**

Dr. Mohamed Gomaa

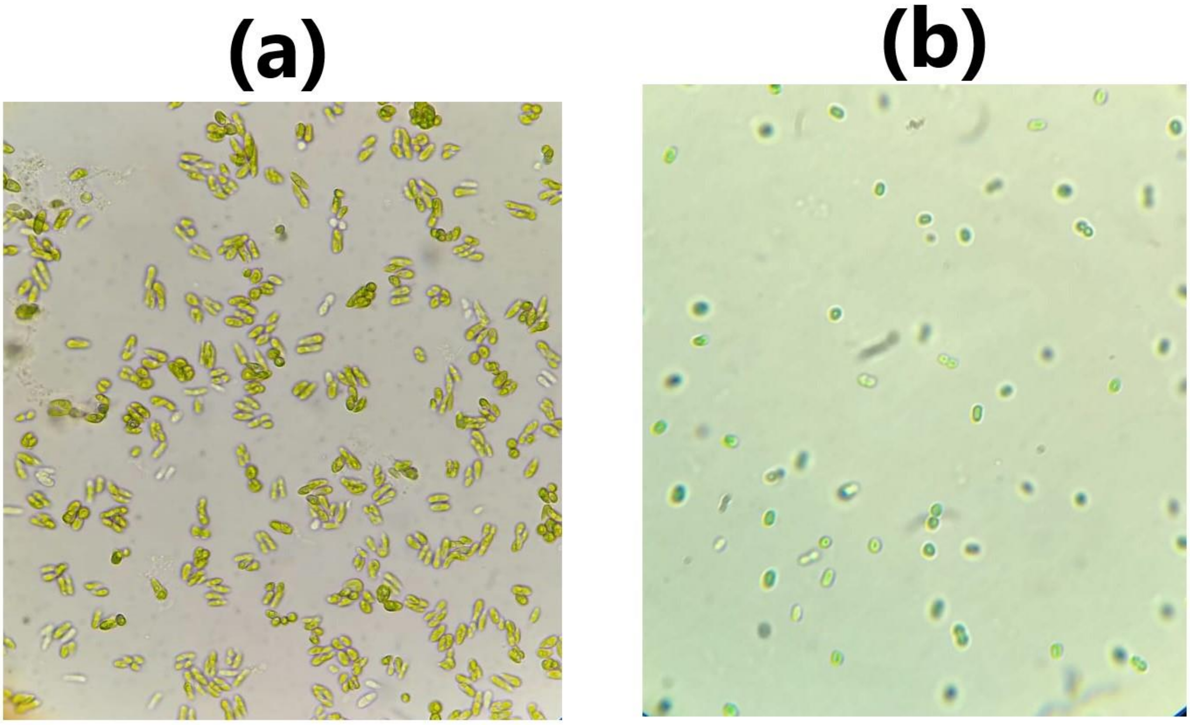

Fig. S1: Photomicrograph of investigated microalgae. (a) *Tetradesmus obliquus,* (b) *Cyanothece* sp.

Table S1: Chemical composition (gL^{-1}) of clarified cheese whey

**Parameter**	**Concentration**
Reducing sugars	6.67
Total sugars	35.12
Protein	1.56
Lipid	0.07

Table S2:

Quadratic model equations showing the relationship between the investigated responses and the process variables along with the coefficient of determination (R^2), adjusted R^2 and significance for each predictive model.

Alga	Predictive model	R^2	Adjusted R^2	F-value	P-value
T. obliquus	Biomass productivity (mg L^{-1} day^{-1}) = $-243.90 - 32.49A + 61.42B + 72.77C + 3.85AB - 6.15AC - 4.60BC + 0.84A^2 - 3.54B^2 - 5.28C^2$	0.8901	0.8489	21.6047	<0.0001
	Lipid content % = $-219.45 + 15A + 42.27B + 66.63C - 0.77AB - 6.72AC - 7.37BC + 0.24A^2 - 1.76B^2 + 5.12C^2$	0.9389	0.916	40.9972	<0.0001
	Carbohydrate content % = $-97.43 + 16.53A + 20.24B + 15.71C - 0.50AB - 4.07AC + 0.57BC - 1.38A^2 - 1.16B^2 - 6.62C^2$	0.8593	0.8066	16.2893	<0.0001
	Protein content % = $19.25 + 3.34A - 2.58B + 22.32C - 0.67AB + 0.03AC - 1.27BC + 0.90A^2 + 0.25B^2 - 5.10C^2$	0.8951	0.8557	22.7475	<0.0001
Cyanothece sp.	Biomass productivity (mg L^{-1} day^{-1}) = $-575.09 + 44.15A + 99.81B + 213.70C - 1.95AB - 5.12AC - 12.75BC - 2.86A^2 - 4.57B^2 - 38.66C^2$	0.9646	0.9513	72.5618	<0.0001
	Lipid content % = $237.04 - 7.99A - 45.91B - 4.69C - 0.81AB + 1.03AC + 0.91BC + 3.24A^2 + 2.44B^2 - 3.25C^2$	0.8971	0.8586	23.2594	<0.0001
	Carbohydrate content % = $-168.28 + 0.31A + 36.35B + 38.97C + 0.57AB + 0.19AC - 2.05BC - 1.41A^2 - 2B^2 - 6.27C^2$	0.9012	0.8642	24.3278	<0.0001
	Protein content% = $-25.32 - 1.65A + 12.26B - 9.09C - 0.22AB + 0.63AC + 3.38BC + 0.24A^2 - 0.91B^2 - 9.03C^2$	0.86	0.8074	16.3757	<0.0001

A: Cheese whey

B: pH

C: $NaNO_3$

Table S3: Fatty acids' composition and their percentages in the investigated microalgae under the optimized conditions

**Fatty acids**	**Code**	***T. obliquus***	***Cyanothece* sp.**
Decanoic acid (n-Capric acid)	C10:0	-	13.40
2-oxooctadecanoic acid	2-oxo-C15:0	1.12	-
Hexadecenoic acid (palmitic acid)	C16:0	53.92	70.68
Octadecanoic acid (stearic acid)	C18:0	-	65.98
9-Octadecenoic acid (oleic acid)	C18:1	18.08	10.31
cis-Vaccenic acid	C18:1	14.56	10.31
trans-13-Octadecenoic acid	C18:1	8.32	-
cis-10-Nonadecenoic acid	C19:1	2.08	-
2-Octyl-cyclopropanedodecanoic acid	13,14-cpa-C23:0	1.92	-
% Saturated fatty acids		56.96	79.38
% Monounsaturated fatty acids		43.04	10.31
% Polyunsaturated fatty acids		0.00	0.00

Table S4: Comparison between the biomass productivity and biochemical composition of microalgae under investigation compared to other microalgae from literature under mixotrophic cultivation using cheese whey as an organic carbon source.

Microalga	Cheese whey (CW) (substituted medium)	pH	Light intensity	Biomass	Lipid		Carbohydrate		Total protein		Ref.
				$mgL^{-1}day^{-1}$	%w/w	$mgL^{-1}day^{-1}$	%w/w	$mgL^{-1}day^{-1}$	%w/w	$mgL^{-1}day^{-1}$	
Chlorella vulgaris	CW 100%	-	370 µE m^{-2} s^{-1} (12:12 h)	52.0	20	-	<30	-	<50	-	(Salati et al. 2017)
Chlorella vulgaris	CW (10 g L^{-1} lactose) (BBM)	6.8	72 µmol $m^{-2}s^{-1}$	12.00	-	-	-	-	-	-	(Melo et al. 2018)
Desmodesmus sp.	15% v/v (50% BBM)	7.0	5000 lux (continuous)	34.50	32.83	-	31.88	-	17.43	-	(Salah et al. 2023)
Chlorella sp.	40% v/v CW (BG-11)	7.4	70 µmol $m^{-2}s^{-1}$ (12:12 h)	70	<25	-	-	-	-	-	(Mondal et al. 2016)
Chlamydomonas sp.	40% v/v CW (BG-11)	7.4	70 µmol $m^{-2}s^{-1}$ (12:12 h)	40	38.36	-	-	-	-	-	
T. obliquus	3.5% v/v CW (synthetic medium)	10	48.4 µmol $m^{-2}s^{-1}$	48.69	42.39	20.64	14.45	7.02	22.53	10.97	**This study**
Spirulina platensis	5% v/v CW (Zarrouk's medium)	9.4	238 µmol $m^{-2}s^{-1}$ (12:12 h)	17.93	-	2.56	47.83	-	44.56	-	(Pereira et al. 2019)
Cyanothece sp.	4.5% v/v CW (synthetic medium)	9	48.4 µmol $m^{-2}s^{-1}$ (continuous)	52.78	21.65	11.42	8.14	4.31	14.95	7.89	**This study**
Cyanothece sp.	2.5% v/v CW (synthetic medium)	9	48.4 µmol $m^{-2}s^{-1}$ (continuous)	49.21	5.33	2.62	16.43	8.10	17.82	8.77	**This study**

- Not reported

Table S5: Comparison between main fatty acids in the lipid profile of microalgae under investigation compared to other microalgae from literature under mixotrophic and autotrophic conditions.

Species	Culture conditions	C16:0	C18:1	∑SFAs	∑MUFAs	∑PUFAs	Ref.
Tetradesmus obliquus	Mixotrophic (3.5% v/v cheese whey in synthetic medium, pH 10)	53.92	40.96	56.96	43.04	ND	**This study**
Scenedesmus obliquus	Mixotrophic (40% v/v cheese whey in BBM)	21.7	29.3	25.1	38.0	36.9	(Girard et al. 2014)
***Desmodesmus* sp.**	Mixotrophic (15% cheese whey and 50% BBM)	25.86	37.51	39.01	39.23	21.76	(Salah et al. 2023)
T. obliquus	Mixotrophic (0.5 g L^{-1} N-acetyl glucosamine in BBM)	66.06	ND	90.60	ND	9.40	(Gomaa and Ali 2021)
T. obliquus	Autotrophic (BBM)	54.35	30.29	68.14	30.29	1.57	(Gomaa et al. 2022)
T. obliquus	Autotrophic (BBM + 4 g L^{-1} $NaHCO_3$)	55.57	17.86	75.16	21.13	3.72	(Fawzy et al. 2022)
T. obliquus	Autotrophic (BG-11)	34.26	21.85	34.26	42.71	15.87	(Ahiahonu et al. 2022)
***Cyanothece* sp.**	Mixotrophic (2.5% v/v cheese whey in synthetic medium, pH 9)	65.98	20.62	79.38	20.62	ND	**This study**
***Aphanocapsa* sp.**	Mixotrophic (0.5 g L^{-1} N-acetyl glucosamine in BBM)	72.07	1.75	86.03	1.75	12.22	(Gomaa and Ali 2021)

SFAs: saturated fatty acids, MUFAs: mono-unsaturated fatty acids, PUFAs: polyunsaturated fatty acids.

References

Ahiahonu EK, Anku WW, Roopnarain A, Green E, Govender PP, Serepa-Dlamini MH (2022) Bioresource potential of Tetradesmus obliquus UJEA_AD : critical evaluation of biosequestration rate, biochemical and fatty acid composition in BG11 media. J Chem Technol Biotechnol 97:689–697. https://doi.org/10.1002/jctb.6951

Fawzy MA, El-Naeb EH, Hifney AF, Adam MS, Gomaa M (2022) Growth behavior, phenol removal and lipid productivity of microalgae in mixotrophic and heterotrophic conditions under synergistic effect of phenol and bicarbonate for biodiesel production. J Appl Phycol 34:2981–2994. https://doi.org/10.1007/s10811-022-02845-5

Girard J-MM, Roy M-LL, Hafsa M Ben, Gagnon J, Faucheux N, Heitz M, Tremblay R, Deschênes J-SS (2014) Mixotrophic cultivation of green microalgae Scenedesmus obliquus on cheese whey permeate for biodiesel production. Algal Res 5:241–248. https://doi.org/10.1016/j.algal.2014.03.002

Gomaa M, Ali MMA (2021) Enhancement of microalgal biomass, lipid production and biodiesel characteristics by mixotrophic cultivation using enzymatically hydrolyzed chitin waste. Biomass and Bioenergy 154:106251. https://doi.org/10.1016/J.BIOMBIOE.2021.106251

Gomaa M, El-Naeb EH, Hifney AF, Adam MS, Fawzy MA (2022) Coupling phenol bioremediation and biodiesel production by Tetradesmus obliquus: Optimization of phenol removal, biomass productivity and lipid content. South African J Bot 151:604–613. https://doi.org/10.1016/j.sajb.2022.10.044

Melo RG de, Andrade AF de, Bezerra RP, Correia DS, Souza VC de, Brasileiro-Vidal AC, Viana Marques D de A, Porto ALF (2018) Chlorella vulgaris mixotrophic growth enhanced biomass productivity and reduced toxicity from agro-industrial by-products. Chemosphere 204:344–350. https://doi.org/10.1016/j.chemosphere.2018.04.039

Mondal M, Ghosh A, Sharma AS, Tiwari ON, Gayen K, Mandal MK, Halder GN (2016) Mixotrophic cultivation of Chlorella sp. BTA 9031 and Chlamydomonas sp. BTA 9032 isolated from coal field using various carbon sources for biodiesel production. Energy Convers Manag 124:297–304. https://doi.org/10.1016/j.enconman.2016.07.033

Pereira MIB, Chagas BME, Sassi R, Medeiros GF, Aguiar EM, Borba LHF, Silva EPE, Neto JCA, Rangel AHN (2019) Mixotrophic cultivation of Spirulina platensis in dairy wastewater: Effects on the production of biomass, biochemical composition and antioxidant capacity. PLoS One 14:e0224294. https://doi.org/10.1371/journal.pone.0224294

Salah A, Sany H, El-Sayed AE-KB, El-Bahbohy RM, Mohamed HI, Amin A (2023) Growth Performance and Biochemical Composition of Desmodesmus sp. Green Alga Grown on Agricultural Industries Waste (Cheese Whey). Water, Air, Soil Pollut 234:770. https://doi.org/10.1007/s11270-023-06780-0

Salati S, D’Imporzano G, Menin B, Veronesi D, Scaglia B, Abbruscato P, Mariani P, Adani F (2017) Mixotrophic cultivation of Chlorella for local protein production using agro-food by-products. Bioresour Technol 230:82–89. https://doi.org/10.1016/j.biortech.2017.01.030
